# Compressive sensing for spatial and spectral flame diagnostics

**DOI:** 10.1038/s41598-018-20798-z

**Published:** 2018-02-07

**Authors:** David J. Starling, Joseph Ranalli

**Affiliations:** 1 Division of Science, Penn State University, Hazleton, PA 18202 USA; 2 College of Engineering, Penn State University, Hazleton, PA 18202 USA

## Abstract

Combustion research requires the use of state of the art diagnostic tools, including high energy lasers and gated, cooled CCDs. However, these tools may present a cost barrier for laboratories with limited resources. While the cost of high energy lasers and low-noise cameras continues to decline, new imaging technologies are being developed to address both cost and complexity. In this paper, we analyze the use of compressive sensing for flame diagnostics by reconstructing Raman images and calculating mole fractions as a function of radial depth for a highly strained, N_2_-H_2_ diffusion flame. We find good agreement with previous results, and discuss the benefits and drawbacks of this technique.

## Introduction

Engines that operate on combustion remain important prime movers in numerous practical energy applications. Mindful of growing awareness of the role that human sources play in climate change and other environmental hazards, modern research strives to create combustion technologies that produce the smallest environmental footprint possible. To that end, optical diagnostics are employed in combustion research, allowing quantitative observation of a number of flame properties; these include species concentration, temperature, flow velocity and rate of heat release. However, advanced combustion diagnostics often require sophisticated equipment (e.g. high energy lasers, precise timing and gated cameras), whose cost may present a barrier to entry to the field of combustion measurement.

In order to address these cost concerns, we consider here the use of compressive sensing (or compressive sampling, CS)^1–3^ as an alternative diagnostic technique for combustion research. The use of CS allows one to compress a signal (e.g., an image) *during* the acquisition process, rather than after the signal is acquired. In general, this allows one to shorten the acquisition time relative to the Nyquist rate^4^. Additionally, CS typically collapses multidimensional data (e.g., a 2D image) into a series of point measurements (e.g., number of photons). For images, this is commonly referred to as a single-pixel camera^5^. In general, this allows one to reduce experimental complexity. This technique has been applied to fields ranging from high-dimensional entanglement^6,7^, hyper-spectral imaging^8^, spectroscopy^9,10^ low-light LIDAR^11^, spatial multiplexing cameras^12^ and biological microscopy^13^. With these advances in novel sensing techniques, compressive sensing may allow for similar or related measurements to be made using inexpensive equipment, lowering the cost barrier for advanced combustion diagnostics in small laboratory settings. However, employing compressive sensing for these purposes presents additional limitations that researchers must consider. These limitations will be discussed later in the Discussion and Conclusion sections.

In this paper we demonstrate the application of CS for reconstructing spatially dependent Raman spectra of four different species in a single flame. We use data from a highly strained diffusion flame with an N_2_-H_2_ fuel mixture probed with a 1 J, 532 nm Raman pump. Raman data was collected via the traditional method from which we simulate the effect of CS. We provide evidence that similar spectra can be obtained using a low cost CW laser, a digital micro-mirror device^14^, and one or two low noise detectors. In addition to the reduced cost of this design, CS also has improvements over point-wise measurement schemes. In particular, CS allows for faster data acquisition for low-light level applications with no moving parts and effectively reduces Gaussian noise during reconstruction.

In the following section, we discuss the specifics of compressive sensing, followed by its application to flame diagnostics. We then present our results, followed by a conclusion.

## Compressive Sensing

Compressive sensing is a technique where an experimenter acquires a signal **x** in such a way as to take advantage of the sparsity of **x**^15^. That is, if **x** has a representation in some basis where only a few appreciable elements exist (and the rest can be ignored), it is considered sparse and CS can be employed. As an example, if one wishes to acquire a signal **x**(**t**) that is a combination of sinusoids, then its discrete Fourier transform $\hat{{\bf{x}}}{\boldsymbol{(}}{\bf{f}}{\boldsymbol{)}}$ has only a few non-zero components and is therefore sparse in that basis. This can be useful for images that have only a limited number of non-zero components–such as an astro-photograph–and most of the image is black. Compare this to a typical 2D image compressed using the discrete cosine transform (e.g., JPEG compression); in such an example, the signal must be transformed to a new basis before it can be sparsely represented, and then transformed back to its original basis. For the purposes of this work, we consider 2D spectral images in the pixel basis and no transform is required.

Let us assume that the size of the discrete signal **x** is an integer *N*. For simplicity, we assume that **x** is a 1-D vector of length *N*; however, the signal can in principle be an array of any dimension. Once we have established that our unknown signal is sparse (note that we do not need a transformation), we take $M\ll N$ measurements, represented by an *M* × *N* sensing matrix **A**. The resulting operation is simple:

1 $${\bf{y}}={\bf{A}}\cdot {\bf{x}}{\rm{.}}$$

**Figure 1:**
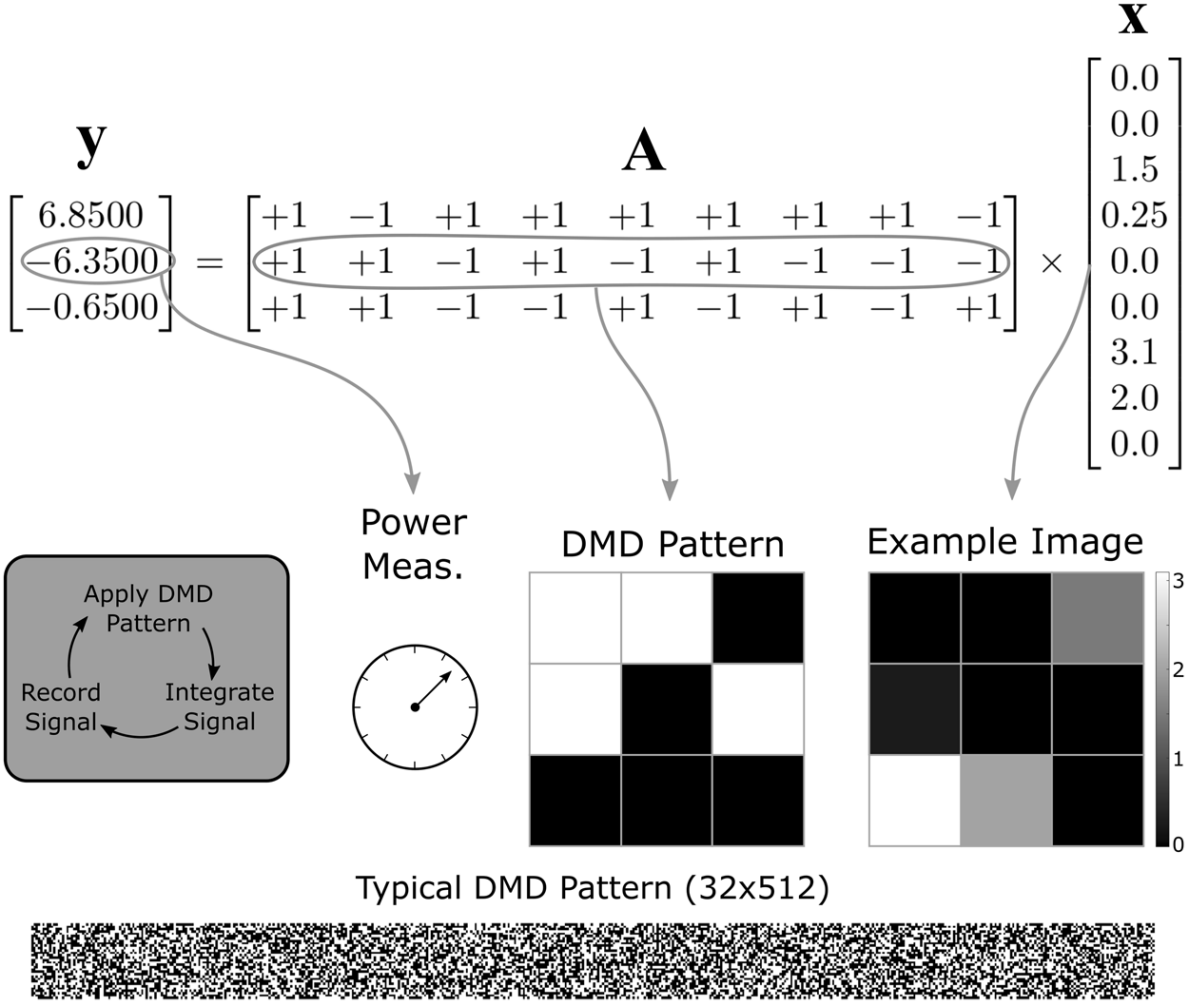
Visualization of compressive sensing. For clarity, we consider a 3 × 3 example image (*N *= 9) with a 33% measurement rate (*M* = 3). This image is 4-sparse, i.e., has only four non-zero elements. The image is flattened to 1D and represented as the length-9 vector **x**. The 3 × 9 measurement matrix is a random sequence of ±1s; each row corresponds to a pattern on the DMD. Black pixels on the DMD represent light going to detector 1, while white pixels represents light going to detector 2. The measurement result is an element in vector **y** and is obtained physically by subtracting the power incident upon detector 1 from the power incident upon detector 2. If only one detector is used, the sensing matrix becomes a series of 1s and 0s instead. In an experiment, **y** is measured, **A** is known, and **x** is found via reconstruction. A typical DMD pattern is shown for the $32\times 512$ Raman images analyzed.

Thus each row in **A** samples **x** and returns a single value, stored in the *M*-length measurement vector **y**. The sensing matrix can take any form such that **A** is any *M* × *N* real matrix; however, we consider a sensing matrix with each cell taking a value of ±1s. In particular, we construct **A** by pulling random columns from a randomly row-permuted *N* × *N* Hadamard matrix^16,17^. This standard procedure ensures fair sampling and allows for fast reconstruction due to fast Hadamard transforms^18,19^. A low-dimensional schematic of this process is shown in Fig. 1; additionally, we will discuss the physical meaning of the sensing matrix and the resulting measurement vector in the following section.

If we wish to acquire the unknown signal **x** (*N* measurements), we can instead physically apply the sensing matrix **A** to **x** one row at a time, obtain $M\ll N$ measurement results and store them in the vector **y**, and finally invert equation 1 to solve for **x**. This measurement reduction is the main advantage of CS, with added mathematical complexity of inverting the sensing matrix **A**. In particular, this is an under-determined linear algebra problem. Therefore, we must apply some constraints; namely, that **x** is *k*-sparse:

2 $$| | {\bf{x}}{| | }_{0}=\sum _{i}| | {x}_{i}{| | }^{0} < k{\rm{.}}$$

That is, the number of non-zero elements in **x** is less than some small number $k\ll N$^15^. We can therefore solve equation 1 subject to minimizing ||**x**||_0_. This is the original application of CS^1,2^.

However, for most natural images, it is more effective to solve equation 1 by minimizing the total variation TV(**x**) of the image^20,21^.

3 $$\mathop{{\rm{\min }}}\limits_{{\bf{x}}\in {{\mathbb{R}}}^{N}}[\frac{\mu }{2}{({\parallel {\bf{y}}-{\bf{A}}\cdot {\bf{x}}\parallel }_{2})}^{2}+{\rm{TV}}({\bf{x}})],$$

where *μ* is a constant regularization factor and $| | \star {| | }_{2}$ is the ${\ell }_{2}$ norm. The total variation of **x** is the sum of derivatives defined by:

4 $${\rm{TV}}({\bf{x}})=\sum _{i}| | {D}_{i}{\bf{x}}{\rm{| | .}}$$

In equation 3, the first term represents the error between the the measurements (**y**) and our sample of the reconstructed signal (**A **· **x**). The second term ensures that our signal is sparse in its derivative. To solve equation 3, we employ the Total Variation Minimization by Augmented Lagrangian and Alternating Direction Algorithm (TVAL3) developed by Li, Yin and Zhang^21^. In particular, we use the anisotropic TV/L2 + model, which requires the signal **x** to be positive and takes the ${\ell }_{1}$ norm of the total variation, as shown in equation 4. The algorithm minimizes an augmented Lagrangian (see equation A-1 in the TVAL3 user guide^21^) by alternately minimizing the first and second terms in equation 3 using the gradient descent method.

In the following section, we will discuss how to implement CS, specifically for flame diagnostics, by utilizing a low-cost digital micro-mirror device (or spatial light modulator).

## Compressive Sensing for Flame Diagnostics

A compressive sensing apparatus is able to replace the role of a camera for data collection with the caveat that only “stationary” signals **x** are able to be measured (discussed further in the Simulation section). There are a variety of combustion diagnostic applications for which this would be applicable. In principle, any combustion diagnostic techniques based upon spontaneous or laser excited emission/scattering of light could utilize compressive sensing approaches. Depending upon the nature of the sensor chosen, it is possible to detect the low intensity light sources commonly required for combustion diagnostics. However, unlike performing diagnostics using traditional amplified imaging tools, such as ICCD cameras, compressive sensing allows for the use of a less expensive single-point sensor, such as an avalanche photo-diode (APD) or photomultiplier tube (PMT). This comes at the expense of losing the ability to take time-resolved or extremely short duration images (due to the acquisition time required by CS), but may still be adequate for some applications, and is preferable to being unable to make a measurement.

Examples of diagnostics that utilize imaging sensors are numerous. Chemiluminescence of various species (CH*, OH*, CO2*^22^) can be imaged to visualize the localized heat release rate distribution. Likewise, while it is more common to perform single-shot laser-induced fluorescence (LIF) imaging, time-averaged LIF images of various species could be obtained in this fashion as well. An example application would be OH planar-LIF, which can be used to visualize the time-averaged position of the flame front. A second type of imaging diagnostic involves images obtained using a spectrometer that are only one-dimensional in space, but contain spectral information. For example, spatially resolved images of spontaneous Raman scattering can be used to generate linear profiles of major species concentration and flame temperature^23,24^. This type of approach could also be useful for performing multi-color pyrometry on sooting flames.

The requirement that compressive sensing produce a single image from a temporal series of samples is limiting in its inability to measure time-varying signals, or to acquire temporal statistical information. Given that many combustion environments of practical interest can be said to be turbulent, this limitation is worth further discussion. Turbulence inherently introduces time dependent behavior on the flame. In principle, two possible experimental approaches to compressive sensing, discussed in the Discussion section, could still allow average values for turbulent signals to be acquired.

**Figure 2:**
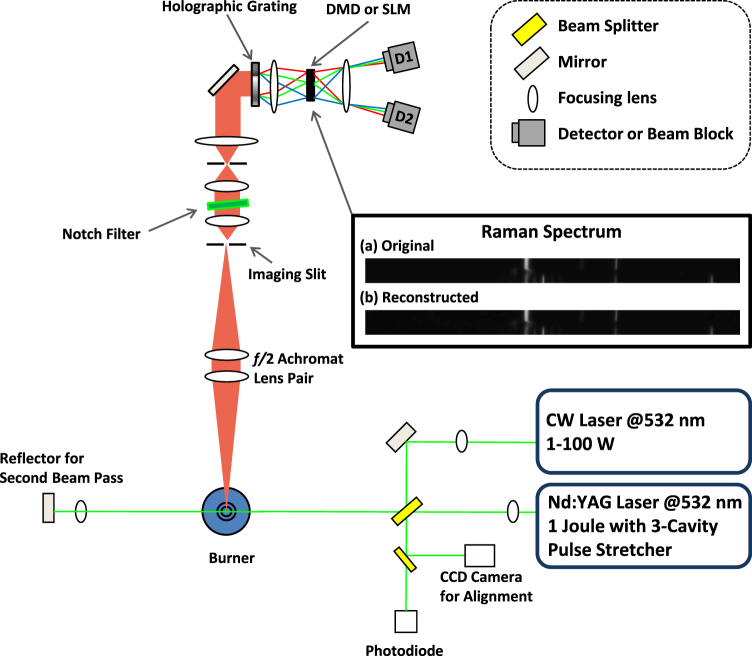
Experimental setup. Either a pulsed Nd:YAG laser or a high power CW diode laser is double-passed through the sample flame. Inelastically scattered Raman light is collected via a lens pair and directed toward a very narrow imaging slit and then through a transmission spectrometer composed of a holographic notch filter, imaging lenses and a holographic grating. The output from the spectrometer passes through a DMD or SLM and the resulting light is directed to two APD (or PMT) detectors with fast timing resolution and high quantum efficiency. Alternatively, one of the detectors can be replaced by a light-dump to reduce cost and complexity. For the SLM, polarization optics would be required to separate the two paths. The inset shows the (**a**) original and (**b**) reconstructed Raman spectrum measured 5.5 mm from the nozzle. The 512 × 32-pixel image has been scaled for clarity and shows the long axis as the spectrum and the short axis as the radial depth.

In the present work, we consider spatially resolved spontaneous Raman scattering. We use data collected in a previous experiment (see Discussion section) and simulate the effect of applying CS. To do so, we introduce a low-cost digital micro-mirror device (DMD) or a spatial light modulator (SLM) and one or two low-noise point detectors (e.g., APDs or PMTs), as shown in Fig. 2. The DMD is an array, similar to a CCD, but each pixel is composed of a controllable mirror that can direct light in two different directions: toward detector 1 or toward detector 2 (or a light-dump). A SLM, on the other hand, can induce a pixel-dependent phase shift which, with a set of polarizing optics, can redirect light incident on a pixel to one of two detectors. In each case, the experimenter has the ability to separate an image into two distinct paths, pixel-by-pixel. The use of two detectors can shorten acquisition time, since both positive and negative elements of the sensing matrix can be acquired simultaneously in each path; however, a single detector can still acquire the same data by first detecting the positive elements and then detecting the negative elements. This would increase the acquisition time by a factor of 2, but would reduce cost and complexity.

We say that the number of pixels on the DMD is *N*, which is equivalent to the number of columns in the sensing matrix **A**. For a sensing matrix composed of ±1s, each mirror sends all the light (with no attenuation) to one of two detectors according to the entry in **A**. We then subtract the total number of photons striking detector 1 from the total number of photons striking detector 2 (or *vice versa*) for each of the $M\ll N$ measurements. For a sensing matrix composed of zeros and 1s, we simply add up the total number of photons striking detector 1 and ignore the light striking a light-dump, for each of the $M\ll N$ measurements. The former method is preferable for a variety of reasons, including reducing noise and acquisition time. As another example, for a sensing matrix composed of +1s and −1/2s, we would direct all the light from some pixels to detector 1, and half of the light from the rest of the pixels to detector 2, and then subtract the values to obtain the measurement result. Any values can be used in **A**, which provides experimenters a way to calibrate for any losses in the resulting system. Either way, this process turns the actual image **x** of size *N* into a list of measurements **y** of size *M* via equation 1.

## Results

### Previous Experiment

Simulated compressive sensing was applied to Raman spectroscopy data from a combustion experiment. The experimental setup and analysis are described in detail by Ranalli and Strakey^25^, but the relevant details are summarized here. Ranalli and Strakey generated a highly strained diffusion flame using an N_2_-H_2_ fuel mixture. The flame was pumped with a frequency-doubled neodymium-doped yttrium aluminum garnet (Nd:YAG) laser emitting 8 ns, 1000 mJ pulses centered at 532 nm with a repetition rate of 10 Hz. Due to laser-induced breakdown of the gases, these pulses were stretched to 100 ns to reduce peak power^26^, and then double-passed through the probe volume with a 120 *μ*m beam waist. Inelastically scattered light was collected via an f/2.0 achromatic pair and focused through a chopper and into a Kaiser Holospec f/1.8 transmission spectrometer with 0.15 nm/pixel resolution. Rayleigh scattering was removed by a holographic notch filter, and both Stokes and anti-Stokes Raman spectra were recorded in a single image. The image was obtained with a 2048 × 2048, 16-bit Pixis deep-cooled CCD camera operating at 90% peak quantum efficiency. The image contained spectral information (along one axis) as well as radial flame depth (along the other axis). A shutter and chopper^27^ were used to reduce the integration window to 120 *μ*s per image in order to limit the contribution of stray light.

For read-noise reduction, the images were binned by 4 pixels spectrally and 8 pixels spatially, producing a 512 × 256 image which was then cropped in the original analysis to 512 × 131 to focus on the flame. For the current analysis, we consider only the 32 spatial pixels on one side of the center of the flame, further reducing the image to 512 × 32. This portion of the image contains the majority of the relevant data. In the original experiment, a total of 100 images (with 120 *μ*s exposure) were recorded in series, separated by 1.0 s each. It is from these images that we conduct our analysis.

### Simulation

We first considered the sort of noise we might expect to find in a typical image. As expected, the original images exhibit fluctuations from laser shot to laser shot consistent with detector shot noise, in addition to fluctuations due to flame turbulence. As discussed below, we assume that our measurements average out the turbulent nature of the flame. Therefore, our simulations only add shot noise to each measurement; i.e., normally distributed noise proportional to the square root of the average pixel value. Note that for CS, there is only a single pixel. We should also note here that shot-noise has a limited impact on CS images when compared to CCD images. If we consider each pixel as its own measurement, then each pixel will have its own signal-to-noise ratio (SNR) that is proportional to the square root of the pixel value. For dark pixels, the SNR will be small, and for bright pixels, the SNR will be large. However, for CS, each measurement collects all (or half) of the light and produces the maximum SNR possible for that experimental situation. This can have a positive overall effect on the final reconstructed image. However, it is well known that CS algorithms introduce noise, which can mitigate the improvement in SNR.

The simulation begins by taking an average of the 100 images in the dataset from which CS can be employed. Each exposure has an effective length of 120 *μ*s (12 ms total) due to slow mechanical shuttering and chopping of the signal, and corresponds to a single 1 J laser pulse (100 J total in 100 images). Due to the readout time of the camera, acquisition of 100 images requires 100 s of total data collection.

**Figure 3:**
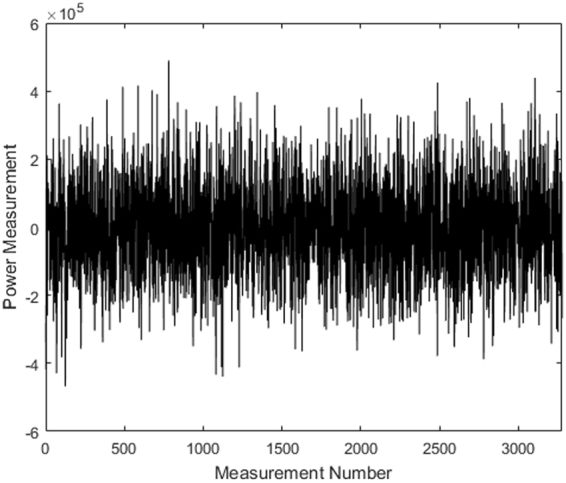
Typical measurement result **y** for a 20% measurement rate. The average value is zero due to the balanced sensing matrix **A**. Fluctuations around zero determine which sparse elements in **x** are significant. The results appear random due to **A** being incoherent with **x**.

The resulting average image is then used as the unknown signal **x** of length $512\times 32={\rm{16,384}}$ for the simulation. Note that this average image includes realistic systematic and statistical errors associated with the experiment. Each element of **x** is sampled with a randomly generated sensing matrix **A** of dimension ${\rm{4,096}}\times {\rm{16,384}}$ (*M* × *N*). The resulting measurement is a 4,096-length vector **y** = **A **· **x** to which we add shot noise; a typical measurement vector is shown in Fig. 3. Note that we choose 25% as the measurement rate of *R* = *M*/*N* since this provides balance between accuracy and speed. However, we discuss reconstruction error as a function of *R* below and show that as low as 20% is acceptable for the current application. Note that the smaller we make *R*, the faster the data acquisition at the cost of accuracy.

Once the simulated measurements have been made, we perform the TVAL3 algorithm (equation 3) via MATLAB with *μ* = 69.3 (primary penalty parameter), *β* = 96 (secondary penalty parameter) and a tolerance of 10^−6^. We then repeat the process 100 times using different, random sensing matrices to obtain statistical variations in the results.

### Reconstructions

**Figure 4:**
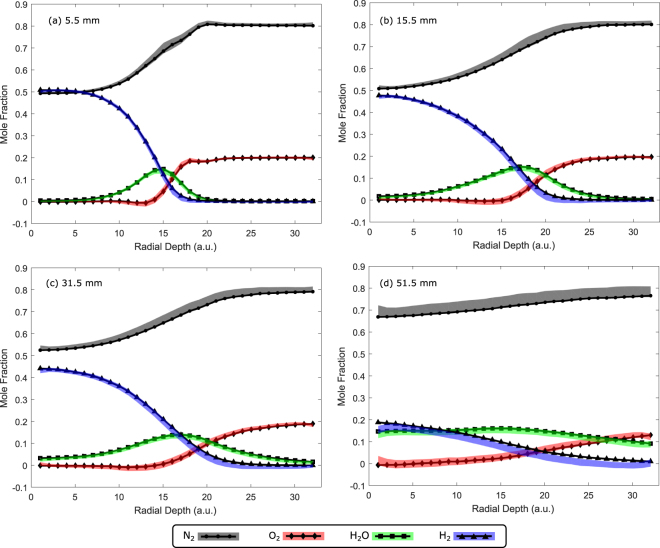
Mole fractions for four species measured at (**a**) 5.5 mm, (**b**) 15.5 mm, (**c**) 31.5 mm and (**d**) 51.5 mm above the nozzle. Black circles (N_2_), triangles (H_2_), squares (H_2_O) and diamonds (O_2_) represent values calculated from an averaged Raman image from the cooled CCD. The gray, blue, green and red bands represent the simulated reconstruction with 25% sampling and a 1-*σ* error band. Radial depth is measured from the center of the flame.

Each reconstructed image is then adjusted as described by Ranalli and Strakey^25^ to correct for optical vignetting and coma. Superpixel values were calculated and a calibration matrix^28^ was applied. Due to the fact that the measurements in this study were simulated, the same calibration matrix that was used in the original experimental study was applied. The original calibration was based upon a laminar flat flame with known equilibrium composition^25^. This resulted in calculations of mole fractions for four species (N_2_, H_2_, H_2_ O and O_2_) for each spatial position. We calculate the mean and standard deviation for the 100 simulations and plot these as error bands against the calibrated average image from the original experiment^25^. These results are shown in Fig. 4.

Note that this is slightly different than the data reduction procedure used in the original work in that the original calibration process was developed around the use of individual shots. Due to this difference, the values achieved here may exhibit some discrepancies relative to the original data. Therefore, one must bear in mind that any experimental setup utilizing CS would require direct calibration appropriate for use on average images. Despite this difference, Fig. 4 demonstrates that CS is capable of producing accurate reduced results when compared to traditional experiments using average images.

Finally, we consider the mean percent error of the mole fractions calculated from the reconstructed signal for each species relative to the calibrated average image as a function of the measurement rate *R*. We define mean percent error as

5 $${\rm{MPE}}=\frac{| | \widetilde{{\bf{x}}}(R)-{\bf{x}}{| | }_{2}}{| | {\bf{x}}{| | }_{2}}\times {\rm{100 \% .}}$$

**Figure 5:**
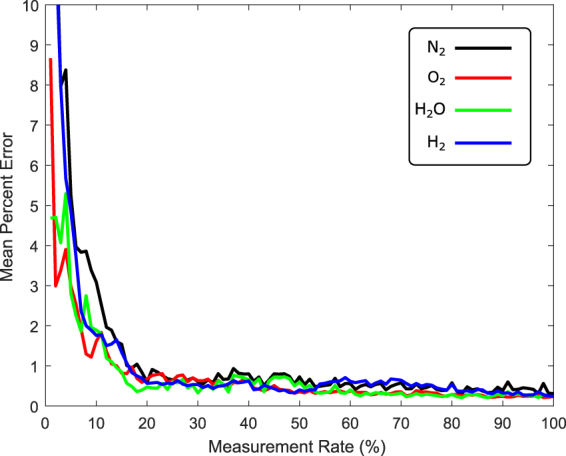
Mean percent error of the mole fractions (calculated from the reconstructed Raman image) relative to the calibrated average Raman image, for N_2_ (black), H_2_ (blue), H_2_O (green) and O_2_ (red) measured 5.5 mm from the nozzle. The measurement rate *M*/*N* refers to the fraction of CS measurements *M* relative to the number of pixels in the image *N*.

Here, $\widetilde{{\bf{x}}}(R)$ is the reconstructed image as a function of the measurement rate, and **x** is the calibrated average image. We see in Figure 5 that for a measurement rate of 20%, the mean percent error for each species, relative to the original results^25^, is less than 1%. This measurement rate of 20% is used in the following section, along with system inefficiencies, to determine feasible acquisition times.

## Discussion

Based upon the results shown in Figs 4 and 5, we see that CS can accurately reproduce Raman spectroscopy data and calibrated mole fractions with minimal errors. These results include all of the systematic and statistical errors of an actual experiment since we begin our simulations with data taken from an experimental system^25^, and include detector shot noise consistent with that data. Additionally, we assume for simplicity that the detection efficiency of the CS detector is equivalent to a deep-cooled CCD; however, in practice, the quantum efficiency of single point detectors (APDs and PMTs) may exceed that of CCDs. We must also consider the added systematic error associated with the DMD (or SLM). In particular, after the imaged flame strikes the DMD (rather than a deep-cooled CCD), light may scatter or get absorbed instead of traversing through the lens toward the detector. The overall transmission probability of a DMD depends upon the specifics of the manufacturer, but we may assume that for a properly aligned DMD and an appropriate anti-reflective lens system for the given detector size, the coupling efficiency should exceed 50%. Therefore, to compare our results to the previous work–which utilized 1 J pulses at 10 Hz–we would require twice as much excitation power (i.e., a 20 W CW laser) to obtain the same signal in the same time. There may also be other effects such as spectrally-dependent scattering which could suppress some wavelengths; however, proper calibration–as in the original work–would account for such limitations.

With these new limitations in mind, we now consider the time of the measurements required in order to produce our results. There are two important time scales: the integration time associated with each of the measurements in **y** and the time to acquire an entire **y** vector, from which we compute a reconstructed image. Because acquiring the required number of measurements in **y** at the 10 Hz repetition rate of the pulsed laser in the original study would be prohibitive, we consider the possibility of utilizing CS with simplified detection hardware (a low-noise point detector) to make this measurement using simplified excitation (a CW laser). As mentioned, the original spectra were measured over approximately 100 s of data collection, due in large part to the readout time of the deep-cooled CCD. However, a typical CS setup requires only a DMD (or SLM) and a low-noise point detector which can have relatively fast timing specifications and very short detector dead time. For example, a typical DMD array that could be used for CS imaging is capable of switching patterns between 20,000 and 30,000 times per second, a minimum switching period of roughly 33 *μ*s. Based upon Fig. 5, we need to utilize a measurement rate of *R* = 20% to achieve 1% accuracy, which requires about 3,277 measurements for the 16,384 pixel image. Given the described physical limitations, this could be achieved in as short a time as 0.11 s. However, the non-stationary nature of a turbulent flame (and power limitations from the excitation laser) makes this acquisition impossible. Two approaches exist by which time-averaged turbulent flames could be imaged using a CS technique: by increasing the individual measurement integration time to ensure smoothing over turbulence for every element in **y**, or by using repeated accumulations of the vector **y** allowing data sampled at non-consecutive timing windows to be utilized. The latter more closely resembles the accumulation of 100 short-exposure images over the course of a longer (100 s) time window as in the original experiment.

To utilize the first approach, while still matching the 100 s acquisition time in the original study, we must make 3,277 measurements over a 100 s period. That is, each measurement should take no longer than 30.5 ms (28.6 Hz). We should note here that we do not have a precise model (or experimental data) describing the turbulence of the flame and therefore cannot rule out turbulence at any frequency. However, this acquisition window would intrinsically provide smoothing of turbulent fluctuations occurring at higher frequencies, allowing time-averaged acquisition to be performed. In principle, measurements could be made with shorter acquisitions (total acquisition time required varies linearly with individual measurement time), but would be limited by the turbulent timescales influencing the measurement. Longer integrations that smooth lower turbulent frequencies would be possible as well, but would extend the acquisition duration.

Using the repeated accumulation approach, we might imagine that all permutations in matrix **A** were tested once per second (about 300 *μ*s per measurement). Each second of data now represents a full measurement of the vector **y**, but would be expected to result in a measurement affected by any flame fluctuations occurring slower than about 3000 Hz. These fluctuations would show up as higher levels of noise scattered throughout the image in the CS reconstruction. Elimination of these fluctuations (and thus the noise) would occur by combining additional accumulations using the subsequent one-second-long windows. This has the advantage that the averaging of the noise could be observed in real-time, allowing the process to be stopped after sufficient accumulations have occurred. Simulated testing of this process using the individual frame images from the original experimental study suggests that the number of accumulations required for this type of averaging would be between 20 and 80, and is a strong function of the distance above the burner tip (and therefore the level of turbulence). Continuing the assumption of completing a sample of the vector **y** every 1 s, this remains within the 100 s acquisition used in the original study, though the potential for a smoothed image to be achieved more quickly exists.

An additional complication arises when considering the effect of chemiluminescence on the ratio between the Raman peaks and the background for these measurements. The initial dataset was based upon 1 J laser pulses that were acquired during an approximate 120 *μ*s acquisition window, during which chemiluminescence was incident on the detector. Since the Raman signal is expected to grow linearly with laser power, maintaining this contrast ratio would be expected to require 1 J compressed into the same time window, or a 8.3 kW CW laser. However, this high power is likely to be unnecessary due to possible improvements over the original design that continuous operation can offer.

In the original experiment, a rotating chopper with a 10 mm slit was used to aperture the the laser’s path through the medium. This large slit (relative to the beam waist) was necessary due to the experimental challenges associated with timing the laser pulse relative to the shutter exposure. For a continuous measurement using CS, a much smaller fixed slit (down to the beam waist of 120 *μ*m) could be used, effectively reducing the chemiluminescence by a factor of 83. We can therefore consider laser energies down to 12 mJ for the same timing window of 120 *μ*s–a 100 W average power laser–and still expect the same signal contrast (albeit without consideration of acquisition noise). Furthermore, even for lower power lasers and long integration times, it may still be feasible to subtract off the small chemiluminescence signal and still get quality results, as more time-averaging would be expected to result in a steadier chemiluminescence background. In fact, we have found that reducing the contrast ratio by simulating an increase of the chemiluminescence by a factor of 100 does not strongly affect the background subtraction or the CS reconstruction. Therefore, even a 1 W laser may be sufficient for this purpose, if increased uncertainty due to the acquisition SNR is acceptable.

Finally, we should consider the approximate cost of the proposed diagnostic. We ignore the burner and optics common to both systems and focus on the replacement parts; i.e., the DMD, the detectors and a broadband anti-reflective lens. At the time of writing, the typical approximate cost for a DMD ranges from $200 (low resolution) to $1,000 (full HD resolution). For the work reported here, only low resolution would be required (e.g., Texas Instrument DLP2000 series). Additionally, low noise biased silicon detectors typically cost approximately $150; for very high sensitivity, PMTs could be used (approximately $2,500). Finally, a ø2.0″ achromatic doublet with anti-reflective coating costs approximately $110. In total, the apparatus requires approximately $460 for low sensitivity and resolution, and $3,610 for high sensitivity and resolution. By contrast, a low-noise CCD (without cooling) costs approximately $3,000, whereas a cooled, intensified CCD with fast timing resolution costs in excess of $70,000.

## Conclusion

We have considered the use of compressive sensing for spatially resolved, spontaneous Raman spectroscopy of a highly strained, N_2_-H_2_ diffusion flame. We used previous data to analyze the efficacy of CS for calculating mole fractions of four species and found close agreement with previous results. Using low-cost equipment, including a DMD, a silicon diode detector, APD or PMT (instead of a deep-cooled CCD) and a CW diode laser (instead of a pulsed Nd:YAG), simulations have shown that equivalent time-averaged results are possible with low error and short acquisition times. These results are consistent with previous work using “single pixel” cameras^5^ for spectroscopy^9,10^. The resulting cost savings range from $2,500–$66,000 depending on the level of sensitivity required. These results rely on the assumption that the average flame can be acquired from 300 *μ*s–30 ms of integration, which is supported by our analysis of previous data. Therefore, CS may provide a valuable alternative for flame diagnostics for small labs with limited funding. Additionally, more advanced video CS schemes, which model the evolution of the image as a linear dynamical system^29^ or use a multiscale video sensing technique^12^, may be used to capture the turbulent nature of the flame. We posit that CS would be worthy of consideration for other combustion diagnostic imaging applications as well, and propose experimental exploration of this technique.

### Data availability

The datasets generated during and analysed during the current study are available from the corresponding author on reasonable request.
